# Copper Inhibits NMDA Receptor-Independent LTP and Modulates the Paired-Pulse Ratio after LTP in Mouse Hippocampal Slices

**DOI:** 10.4061/2011/864753

**Published:** 2011-10-19

**Authors:** Nina L. Salazar-Weber, Jeffrey P. Smith

**Affiliations:** Department of Biology, Colorado State University-Pueblo, 2200 Bonforte Boulevard, Pueblo, CO 81001, USA

## Abstract

Copper misregulation has been implicated in the pathological processes underlying deterioration of learning and memory in Alzheimer's disease and other neurodegenerative disorders. Supporting this, inhibition of long-term potentiation (LTP) by copper (II) has been well established, but the exact mechanism is poorly characterized. It is thought that an interaction between copper and postsynaptic NMDA receptors is a major part of the mechanism; however, in this study, we found that copper (II) inhibited NMDA receptor-independent LTP in the CA3 region of hippocampal slices. In addition, in the CA3 and CA1 regions, copper modulated the paired-pulse ratio (PPR) in an LTP-dependent manner. Combined, this suggests the involvement of a presynaptic mechanism in the modulation of synaptic plasticity by copper. Inhibition of the copper-dependent changes in the PPR with cyclothiazide suggested that this may involve an interaction with the presynaptic AMPA receptors that regulate neurotransmitter release.

## 1. Introduction

Copper is a trace element that plays many important roles in the brain, one of the most copper-rich organs of the body. It is an essential structural component and cofactor for many proteins and enzymes, including various effectors of synaptic plasticity, suggesting that it has an important role in regulating the cellular processes underlying learning and memory [1, 2]. The importance of copper in learning and memory is underscored in neurodegenerative diseases including Alzheimer's, Menkes', Wilson's, and Prion disease, in which misregulation of copper is strongly associated with learning and memory deficits [1, 3–5]. It is estimated that copper is present in the extracellular space of brain tissue at a concentration between 0.2 and 1.7 *μ*M; however, it is released during neurotransmission into some glutamatergic synapses where it transiently reaches levels estimated to rise to as high as a few hundred micromolar [6–8]. Synaptically released copper may regulate synaptic plasticity by dampening NMDA, AMPA, and GABA receptor function, as each of these receptors is inhibited by copper at concentrations ranging from the low nanomolar for GABA receptors, to the low micromolar, for AMPA and NMDA receptors [9–14]. However, very little is currently known about the precise mechanisms by which copper interacts with the cellular processes governing learning and memory.

Doreulee et al. (1997) first reported that long-term potentiation (LTP) in the CA1 region of the rodent hippocampus is inhibited by 1 *μ*M copper (II) when present in the extracellular solution bathing slice preparations [15]. LTP, the activity-dependent strengthening of synaptic communication, is a form of synaptic plasticity that is widely accepted as a major mechanism underlying learning and memory [16, 17]. The inhibition of LTP by copper was later repeated and was also observed in brain slices of rats after chronic ingestion or intraperitoneal injection of copper; however, the mechanism behind the inhibition has not been characterized [18–20]. Investigators have suggested that the mechanism may be NMDA receptor dependent; however, it has not yet been investigated whether inhibition of LTP by copper can occur without a contribution from NMDA receptors. It was also shown in one study using slices from the CA1 region of the hippocampus that copper affected the paired-pulse ratio (PPR), a marker of short-term plasticity that is expressed presynaptically [18]. Therefore, in this study, we investigated the inhibition of NMDA receptor-independent LTP by copper in the CA3 region of mouse hippocampal slices. Complete inhibition was observed, and, because LTP in this region is expressed presynaptically, a presynaptic mechanism was suggested. This was further supported by our additional studies showing that the PPR was modulated by copper in both the CA3 and CA1 regions in a manner that was entirely dependent upon the expression of LTP. These results demonstrate that copper can affect presynaptic function during its modulation of hippocampal synaptic plasticity and, therefore, extends our understanding of its mechanism of action beyond a more simple model that involves only the postsynaptic machinery.

## 2. Materials and Methods

### 2.1. Brain Slice Preparation

Electrophysiological experiments were conducted in accordance with the Colorado State University-Pueblo Institutional Animal Care and Use Committee (IACUC) guidelines essentially as described previously [21]. Briefly, Swiss Webster mice were housed in standard conditions with 12 hours of light and 12 hours of dark. They were given unlimited access to lab chow and tap water that was filtered using a household pitcher-style filter (Brita). Males and females were used at random between the ages of 1 to 3 months. Mice were sacrificed by decapitation, and a Lancer Vibratome Series 1000 was used to make 350 *μ*M transverse brain slices through the hippocampus. The brain slices were incubated for a minimum of one hour in artificial cerebrospinal fluid (ACSF) supplemented with 2 mM ascorbic acid before being transferred to the recording chamber. ACSF contained (in mM) 124 NaCl, 2.5 KCl, 2 MgSO_4_, 2 CaCl_2_, 10 D-glucose, 1.25 NaH_2_PO_4_, and 26 NaHCO_3_ and was bubbled vigorously with 95% O_2_/5% CO_2_. The pH was adjusted with 1 M HCl or 1 M NaOH to between 7.35 and 7.40 and was periodically monitored throughout the course of experimentation. During recording, slices were perfused with oxygenated ACSF at a rate of 3-4 mL per minute at room temperature.

### 2.2. Stimulation Procedure and Statistics

**Figure 1:**
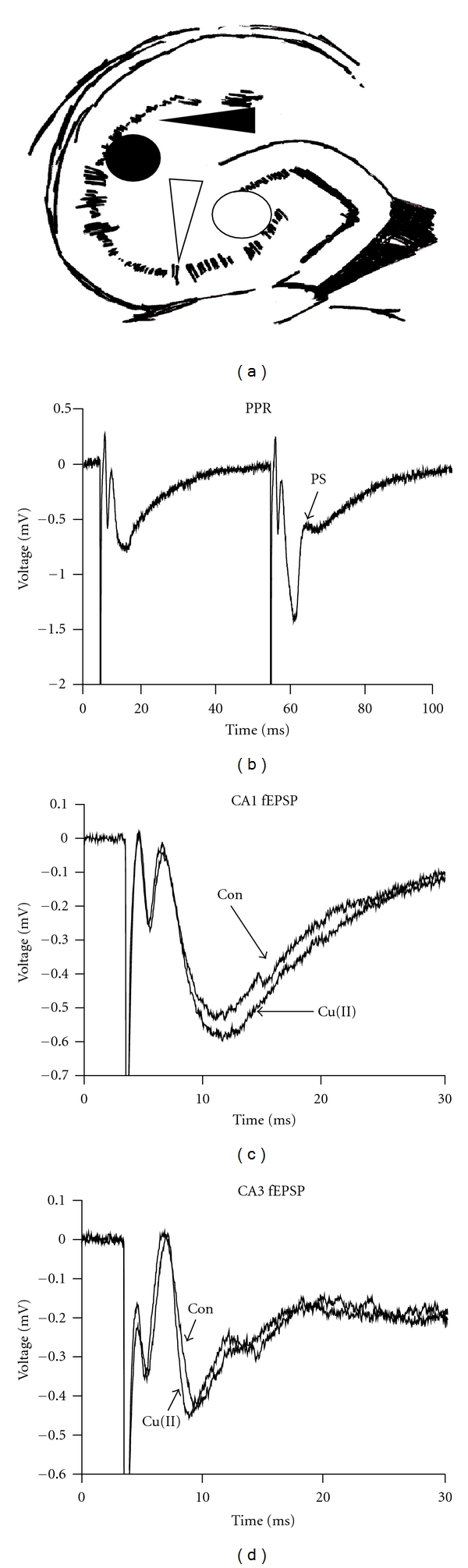
Electrophysiological recording in the mouse hippocampal slice. (a) In the CA1 region, the stimulating electrode (black circle) and the recording electrode (black arrow) were placed in the stratum radiatum. In the CA3 region, the mossy fibers were stimulated (white circle), and recording was done in the stratum lucidum (white arrow). (b) A representative trace from one brain slice showing how a paired-pulse test appeared at the stimulus intensity chosen for recording. (c) and (d) The average of the slopes of the fEPSPs taken 30 seconds before the tetanus was not changed by copper in either the CA1 or CA3 regions (*n* = 8, data not shown). Representative traces from an interleaved experiment are shown.

To measure the field excitatory postsynaptic potential (fEPSP), a 200 *μ*M diameter bipolar concentric stimulating electrode (FHC CBAEC75) and a sharp borosilicate glass recording electrode filled with 2 M NaCl were placed in the stratum radiatum of the CA1 region of the hippocampus at a 250–500 *μ*M interelectrode distance. For CA3 recording, the stimulating electrode was placed on the mossy fiber pathway, and responses were recorded in the stratum lucidum as shown in Figure 1(a). The slice was stimulated with a square pulse of 0.1 ms duration every 30 seconds. To determine the test stimulus intensity, a paired-pulse test was done, and the stimulus intensity was adjusted to the point at which a population spike was evoked on the second, but not the first, pulse as shown in Figure 1(b). This calibration protocol resulted in a stimulus that was 30–50% of the intensity required to elicit a maximum response. The paired-pulse ratio (PPR) was measured at a 50 ms interpulse interval and calculated by dividing the slope of the second fEPSP by the first and converting the ratio to a percentage. For LTP, the baseline fEPSP was obtained for a minimum of 30 minutes, followed by four high-frequency (100 Hz) tetani of one-second duration which were applied in place of the test pulse with a two-minute interval between the second and third tetani. Responses were recorded for 60 minutes after the last tetanus, and the slopes of the fEPSPs were calculated using Clampfit 8 (Axon Instruments). The slopes were averaged over the last five minutes and compared between experimental groups using a paired *t*-test. For the studies with cyclothiazide (CTZ), a two-sample *t*-test was used.

### 2.3. Pharmacological Treatments

Interleaved experiments were conducted with ACSF alone or with 5 *μ*M CuCl_2_, 5 *μ*M ZnCl_2_, 100 *μ*M CTZ, 40 *μ*M picrotoxin, and/or 10 *μ*M MK-801. All test solutions were present for the duration of the experiments and were added directly to the ACSF, except for CTZ and picrotoxin, which were dissolved in DMSO; for these experiments, DMSO was also added to the ACSF for the control slices at a final concentration of 0.1%. All chemicals were purchased from Sigma, except for CTZ (A.G. Scientific, Inc.) and MK-801 (Tocris).

## 3. Results

Extracellular field potential recording was done in the CA1 and CA3 regions of mouse hippocampal slices with and without 5 *μ*M CuCl_2_ in the recording solution for the duration of experiments. The slopes and waveforms of the fEPSPs, taken after a 30-minute baseline, were not significantly different between control and copper-treated slices in either the CA1 or CA3 regions of the hippocampus (Figures 1(c) and 1(d)). This was consistent with previous reports that 1 *μ*M copper (II) did not affect the slope of the fEPSP but that 10 *μ*M copper (II) depressed it to 85% of control [15]. Thus, 5 *μ*M copper did not appear to affect basal synaptic transmission in our experiments.

**Figure 2:**
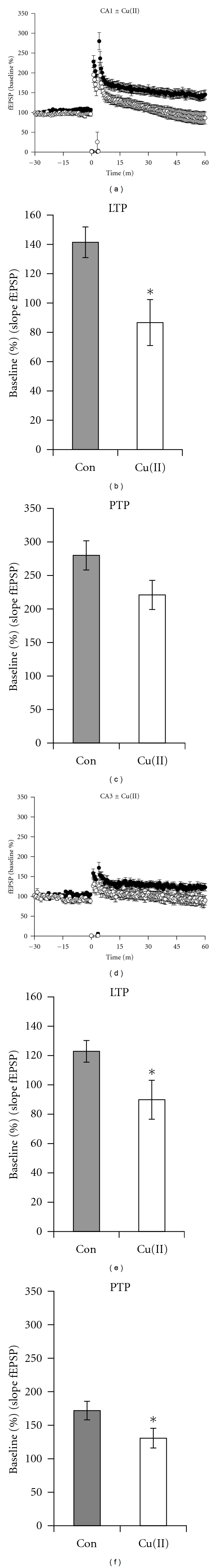
Copper inhibited LTP in the CA1 and CA3 regions of mouse hippocampal slices. (a) In the CA1 region, compared to control slices (black circles), LTP was inhibited by copper (open circles). (b) The average of the fEPSP slopes taken one hour after tetanus was significantly decreased from 141.4 ± 10.5% to 86.6 ± 15.7% in copper-treated slices (*P* = 0.034, *n* = 8). (c) In the CA1 region, the peak PTP was reduced from 280.0 ± 21.8% to 220.9 ± 21.8% in copper-treated slices (*P* = 0.069, *n* = 8). (d) Copper-inhibited LTP in the CA3 region of the mouse hippocampus, control groups are indicated by black circles and copper-treated groups are indicated by open circles. (e) The average fEPSP in the CA3 region measured one hour after tetanus was significantly decreased from 122.9 ± 7.4% to 89.9 ± 13.3% in copper-treated slices (*P* = 0.016*n* = 8). (f) In the CA3 region, the peak PTP was significantly reduced from 171.8 ± 13.9% to 130.8 ± 14.7% in copper-treated slices (*P* = 0.039, *n* = 8).

Confirming previous studies, 5 *μ*M copper (II) completely blocked LTP of the fEPSP slope in the hippocampal CA1 region of our brain slices (Figures 2(a) and 2(b)) [15, 18, 19]. Also, posttetanic potentiation (PTP) in the CA1 region, measured as the peak fEPSP slope immediately following tetanic stimulation, was somewhat reduced in the presence of copper (Figure 2(c)). LTP in this brain region was dependent on NMDA receptors as it was blocked with DAP5 (data not shown). 

To test the requirement for NMDA receptors in the inhibition of LTP by copper, we repeated the above experiments by stimulating the mossy fibers and recording in the CA3 dendritic region with 10 *μ*M MK-801, an NMDA receptor inhibitor, in the bath to isolate NMDA receptor-independent LTP. We were unable to evoke a sufficient LTP to do the experiment in this region; however, with the addition of 40 *μ*M picrotoxin, a GABA receptor inhibitor, robust LTP was present one hour after tetanic stimulation in control slices, and, just as in the CA1 region, copper completely blocked it (Figures 2(d) and 2(e)). In addition, PTP was statistically significantly reduced by copper in the CA3 region (Figure 2(f)). To the best of our knowledge, these results are the first reported demonstrating that copper inhibits NMDA receptor-independent LTP in the CA3 region of the hippocampus. 

**Figure 3:**
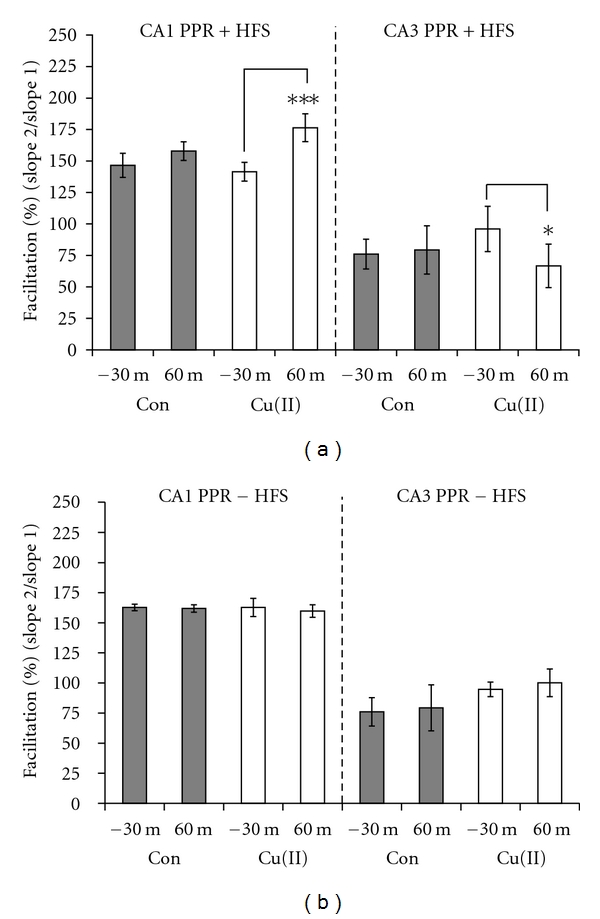
Modulation of the PPR by copper in the CA1 and CA3 regions was dependent on LTP. (a) The average of the PPRs measured 30 minutes before tetanizing the slices was not different in copper-treated (white bars), compared to control slices (gray bars) in either the CA1 or CA3 region. Also, the PPRs in control slices did not change after LTP in either brain region. However, after LTP, copper-treated slices showed a 34.9% enhancement of the PPR in the CA1 region (*P* < 0.001, *n* = 8) and a 29.3% decrease in the PPR in the CA3 region (*P* = 0.05, *n* = 8). (b) Without the HFS, there was no significant change in the PPRs compared between control and copper-treated slices in either the CA1 or CA3 region.

**Figure 4:**
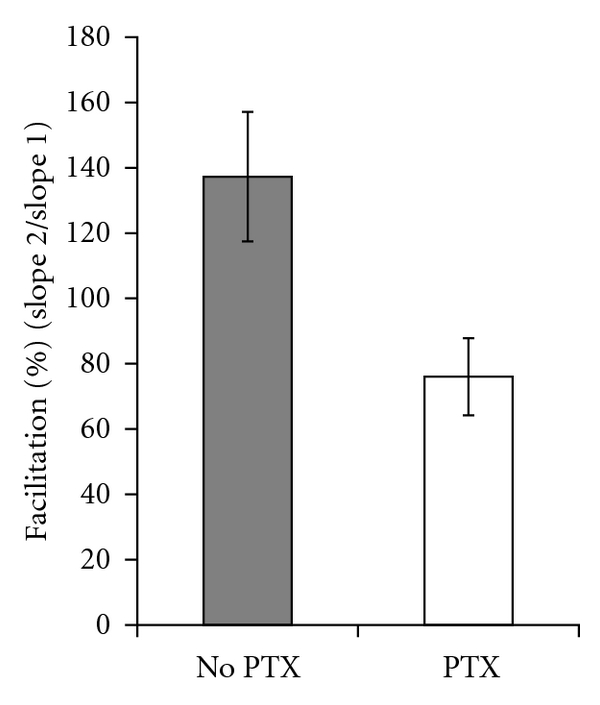
Paired-pulse facilitation was converted to paired-pulse depression by picrotoxin in the CA3 region. The average PPR without picrotoxin was 137.3 ± 19.8% (*n* = 4), but the average PPR with picrotoxin was 76.1%  ± 11.8% (*n* = 8).

Since LTP in the mossy fiber to CA3 cell synapse is thought to be expressed through a presynaptic mechanism [22–25], we were interested in more deeply investigating potential copper-dependent changes in presynaptic plasticity which might occur before and after the induction of LTP. Therefore, we measured the PPR in the presence and absence of 5 *μ*M CuCl_2_. Confirming previous work, copper did not affect the baseline PPR in the CA1 region (Figure 3(a), left panel), and, in the CA3 region, copper only slightly increased it (Figure 3(a), right panel) [15]. With picrotoxin, which as noted was necessary to achieve LTP in the CA3 region, paired-pulse facilitation was converted to paired-pulse depression (Figure 4). To the best of our knowledge, this lack of an effect of copper on the baseline PPR in the CA3 region has not been reported.

Next, we measured the PPR one hour after inducing LTP in the CA1 and CA3 regions and found that the PPR was significantly enhanced in the presence of copper in the CA1 (Figure 3(a), left panel) but significantly decreased in the CA3 region (Figure 3(a), right panel). To determine whether this effect on the PPR was dependent upon LTP and not simply a result of extended exposure to copper, we incubated the slices for 90 minutes in copper without the LTP-inducing high-frequency stimulus, after which there was no change in the PPR in either hippocampal region (Figure 3(b)). Combined, our results in both regions indicated that LTP caused the appearance of a presynaptic sensitivity to copper which was expressed as a modulation of the PPRs. These results extend the findings of Goldschmith et al. (2005) who showed that rats which had chronically ingested copper displayed changes in the PPR following the expression of LTP in the CA1 region [18].

**Figure 5:**
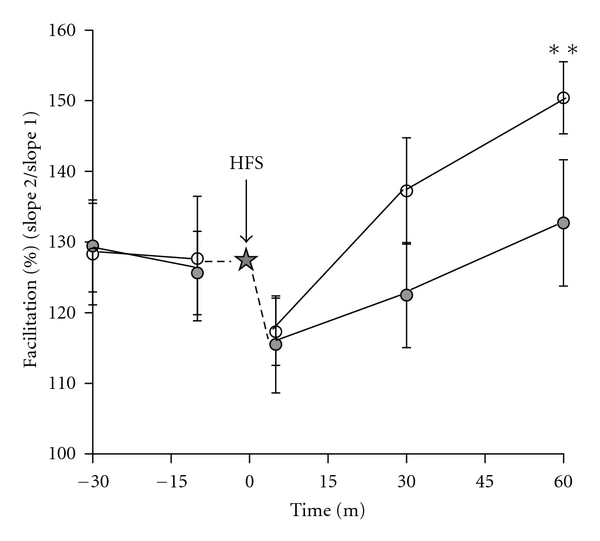
Copper gradually enhanced the PPR after LTP in the CA1 region. Paired-pulse tests were done at intervals before and after the HFS tetanus (star). Prior to the tetanus, the PPR was not significantly different between control slices (filled circles) and copper-treated slices (open circles). Five minutes after the HFS, there was a decrease in the PPR. The difference in the percent facilitation became statistically significant 60 minutes after the tetanus, at which point the average PPR of the copper group was 150.4 ± 5.1% as compared to 132.7 ± 8.9% in the control group (*P* = 0.007, *n* = 5).

The effect of copper on PPRs in the CA1 region appeared gradually, as evident when paired-pulse tests were done at intervals preceding and following the tetanus (Figure 5). Over this time course, we found that the enhancement of the PPR matured and became statistically significant only later, at 60 minutes after tetanus, during the maintenance phase of LTP. 

**Figure 6:**
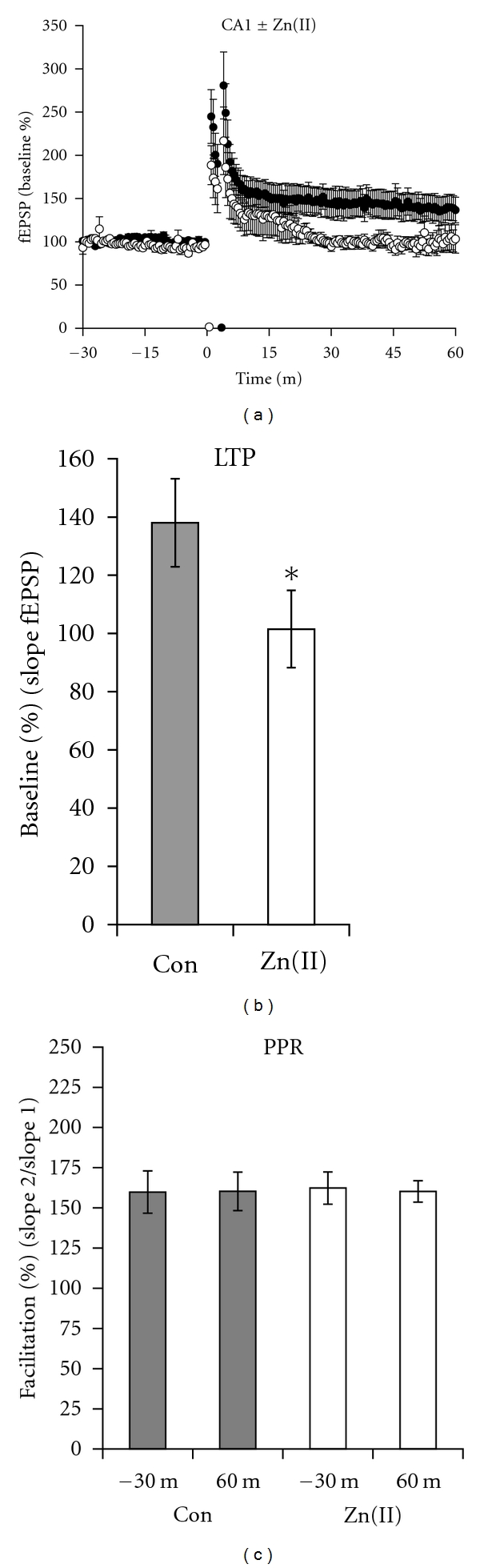
Zinc blocked LTP but did not change the PPR after LTP in the CA1 region. (a) LTP was present in control slices (filled circles) but was blocked by 5 *μ*M ZnCl_2_ (open circles). (b) The magnitude of the LTPs averaged one hour after the tetanus was reduced from 138.0 ± 15.1% to 101.5 ± 13.2% in zinc-treated slices (*P* = 0.013, *n* = 8). (c) The PPR after the expression of LTP was not affected by zinc (open circles). All slices were tetanized at *t* = 0 m in this experiment.

While the effects of copper on synaptic plasticity were clearly NMDA receptor independent in the CA3 region, the NMDA receptor dependence is not as easy to ascertain in the CA1 region. Therefore, to more deeply investigate the potential role of NMDA receptors in the CA1 region, we tested whether the zinc modulatory site of the NMDA receptor was a target for copper [26]. Similar to the work of others who have shown that zinc modulates LTP in the CA1 region, LTP was significantly inhibited by 5 *μ*M ZnCl_2_, just as with copper at an equivalent concentration (Figures 6(a) and 6(b)) [27, 28]. Thus, zinc mimicked the inhibition of LTP by copper. In contrast, zinc and copper had divergent effects on the LTP-dependent changes in the PPR (Figure 6(c)), as there was no significant difference in the PPR before as compared to after LTP in zinc-treated groups. Therefore, copper/LTP-dependent modulation of the PPR in the CA1 region was unique to copper and could not be mimicked by zinc, suggesting the existence of separate mechanisms for the inhibition of LTP and LTP-dependent modulation of PPRs for the two ions.

**Figure 7:**
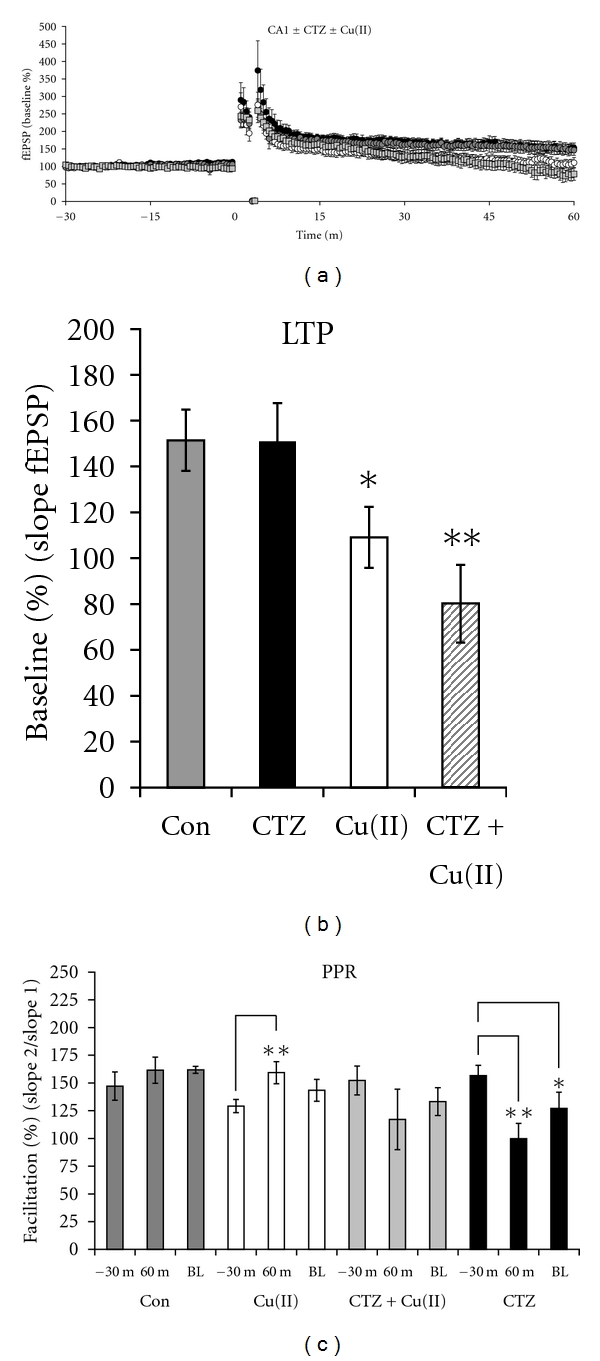
CTZ modulated the effect of copper on synaptic plasticity in the CA1 region. (a) Slices treated with CTZ (black circles) and control slices (gray circles) exhibited identical LTP, but LTP was inhibited by copper (open circles) and by copper combined with CTZ (gray squares). (b) The level of LTP was 151.5 ± 13.4% in control slices (gray bar) (*n* = 8) and 151.0 ± 16.7% in CTZ-treated slices (black bar) (*n* = 8). Copper reduced the LTP to 109.0 ± 13.3% (white bar), and LTP was further reduced to 80.1 ± 17.0% in slices treated with copper and CTZ (striped bar) (*n* = 8). (c) There was no significant difference among the PPRs at 30 minutes prior to the tetanus across groups. At 60 minutes after tetanus, there was no change in the PPR in the control group, a significant enhancement in the PPR in the copper group (*P* = 0.028, *n* = 7), no significant change in the PPR in the CTZ + copper group, and a significant decrease in the PPR in the CTZ group (*P* = 0.006, *n* = 7). The baseline (BL) PPRs measured after 90 minutes without a tetanus was decreased from 152.2 ± 13.1% at 30 minutes prior to the tetanus to 133.3 ± 12.5% in slices treated with CTZ (*P* = 0.018, *n* = 7). The BL PPRs did not vary among the other groups.

Next we examined whether AMPA receptor function might be part of the mechanism by which copper inhibited LTP and enhanced the PPR after LTP in the CA1 region. To do this, we treated brain slices with 100 *μ*M CTZ to inhibit AMPA receptor desensitization. We found that CTZ alone did not alter LTP, consistent with previously published results (Figures 7(a) and 7(b)) [29]. However, CTZ enhanced the inhibitory effect of copper on LTP. The enhancement was synergistic since the effect of both copper and CTZ together reduced the LTP by 71.4% while their individual effects added together only reduced it by 43.0% (Figure 7(b)). Presynaptically, CTZ completely blocked, and even slightly reversed, copper/LTP-dependent enhancement of the PPR without significantly altering the baseline PPRs, suggesting that AMPA receptors might be responsible for the presynaptic effects of copper (Figure 7(c)). However, consistent with previous reports, CTZ alone also significantly reduced the PPR after a 90-minute incubation; therefore, its block of the presynaptic effects was not necessarily LTP dependent and instead may have represented an occlusion of the presynaptic effect (Figure 7(c)) [30, 31].

## 4. Discussion

### 4.1. Copper Inhibits NMDA Receptor-Independent LTP

Inhibition of LTP by copper has been well established in the CA1 region of the hippocampus, but the mechanism remains poorly characterized. It has been suggested that copper inhibits LTP mainly through a postsynaptic interaction with NMDA receptors; however, many other important effectors of synaptic plasticity are known targets of copper which could contribute to the mechanism [15, 18–20]. To clarify this possibility, we showed here for the first time that copper can inhibit LTP independent of NMDA receptors in the CA3 region of the mouse hippocampus. NMDA receptor independence was ensured by the use of 10 *μ*M MK-801, an NMDA receptor antagonist with an IC_50_ of 0.13 *μ*M, and stimulation of the mossy fiber pathway with the recording electrode placed in the stratum lucidum; a placement that has been shown to isolate NMDA receptor-independent LTP in the CA3 region [23, 32].

The inhibition of LTP by copper in the CA3 region of the hippocampus indicated a possible presynaptic mechanism for copper, as LTP in this region of the brain is thought to be expressed through a presynaptic mechanism [22–25]. Since posttetanic potentiation (PTP) is also a presynaptic phenomenon, this was further supported by our observations that copper reduced PTP [33]. Potential non-NMDA receptor presynaptic targets for copper could include the glutamate release machinery since increased release is a major mechanism behind LTP in the CA3 region [25]. While it has not been replicated in intact cells with physiological concentrations of copper, this idea is further supported by studies showing that copper enhances vesicular binding to membrane fractions [34]. Thus, one could speculate that an interaction between copper- and zinc-binding domains on proteins that regulate vesicular release, such as rab3A, could be a specific target of copper [35, 36]. It is also possible that copper may be interacting with presynaptic voltage-gated calcium channels, GABA receptors, Kainate, or AMPA receptors, as each of these has a role in regulating neurotransmitter release [12, 24, 37, 38]. 

CA3 neurons are strongly inhibited by GABAergic pathways, and it was necessary to use picrotoxin, a GABA_A_ receptor inhibitor, in order to obtain LTP in the CA3 region in our studies. GABA receptors serve a complex role in LTP, and there is contradiction in the literature regarding it. For example, in the CA3 region, picrotoxin facilitates LTP, whereas gabazine, used to block presynaptic GABA_A_ receptors, inhibits it [37, 39]. Additionally, copper was shown to inhibit GABA receptors in whole-cell patch clamp studies but acted as an agonist in brain slices [11, 14]. Therefore, the use of picrotoxin in our studies, although necessary, added complexity to interpreting the potential mechanism of the inhibition of LTP by copper in this brain region. Regardless, the major result of this work was the complete inhibition of NMDA receptor-independent LTP in the CA3 region by copper.

NMDA receptors are known to be inhibited by zinc, and, since copper and zinc share similar biological functions in the nervous system, presumably due to their similar valence, size, and charge, we postulated that copper interacts with this site to inhibit LTP [10, 40–42]. In the CA1 region, we showed that zinc inhibited LTP just as copper did, suggesting that copper and zinc may share this mechanism. However, contrary to our results, it was previously demonstrated that zinc positively modulates LTP [43]. This discrepancy with our work could be explained by differences in the stimulus pattern used to induce LTP, as we used four trains at 100 Hz to induce our LTP, whereas one train of 10–100 Hz was used in the previous study [43]. This explanation was further supported in a follow-up publication showing that LTP in brain slices tetanized six times at 100 Hz was not potentiated by zinc, whereas those tetanized once were potentiated [28]. Thus, increasing the stimulus, strength appears to reduce or, in our case reverse, the enhancing effects that zinc exerts on LTP. This is also consistent with studies showing that the LTP-inducing stimulus pattern effects whether the copper-sensitive A*β* peptide of Alzheimer's disease can inhibit LTP [21]. Finally, it should be noted that we performed our studies at room temperature, whereas those which showed that zinc increases LTP were performed at 26-27°C, and this may have contributed in part to the difference [28, 43]. Overall, our work suggested that copper interacts with the zinc-modulatory site on the NMDA receptor as a potential part of the mechanism for inhibition of LTP by copper in the CA1 region of the hippocampus. 

AMPA receptors are a major effector of synaptic plasticity in the hippocampus and are known to be functionally inhibited by micromolar concentrations of copper with kinetics indicative of two binding sites with differing sensitivities to copper [12, 27]. Therefore, we hypothesized that some aspect of AMPA receptor function might explain the inhibition of LTP by copper in the CA1 region. To investigate this, we treated our slices with cyclothiazide (CTZ), an inhibitor of AMPA receptor desensitization, and measured LTP in our system. Consistent with previous studies, CTZ did not significantly affect or mimic the effect of copper on LTP in these experiments [29]. We interpret this to mean that inhibition of LTP by copper was probably not through a mechanism involving desensitization of AMPA receptors. However, because CTZ was synergistic with copper in enhancing the inhibition of LTP, it was suggested that copper might affect it by a mechanism that includes a component of AMPA receptor function. For example, binding to CTZ might induce a conformational change in the AMPA receptor that increases an interaction with copper. 

### 4.2. Copper Affects Presynaptic Plasticity in an LTP-Dependent Manner

The second major finding of the work presented here is that copper modulated the PPR, a measure of the probability of neurotransmitter release (*P*_*r*_), in a strictly LTP-dependent manner in both the CA1 and CA3 regions. These results were consistent with a previously published report showing that the PPR is changed in the CA1 region following LTP in rats that had chronically consumed copper in their drinking water [18]. Our results extended these findings to the CA3 region and suggested that LTP induced a change in the presynaptic terminal that creates or unmasks a sensitivity to copper.

The failure of zinc to affect the PPR after LTP in our experiments in the same way as copper ruled out a mechanism such as an interaction between copper and the zinc-binding domain of presynaptic NMDA receptors. Thus, our studies with zinc indicated that inhibition of LTP and LTP-dependent modulation of the PPR were not expressed by the same mechanism and supported the idea that copper influences synaptic plasticity through multiple mechanisms.

Our result showing that CTZ, a compound that blocks AMPA receptor desensitization, completely blocked the effect of copper on the LTP-dependent enhancement of the PPR in the CA1 region suggested that the presynaptic AMPA receptors which regulate neurotransmitter release may be a primary target of copper. However, because CTZ also reduced the PPR in the absence of copper, the apparent block could have resulted from blocking AMPA receptor desensitization to increase the *P*_*r*_, decrease the PPR, and occlude the copper-dependent increase in the PPR [30, 31]. On the other hand, CTZ and copper could exhibit opposing effects through uniquely different interactions with AMPA receptors. Such opposing effects would be consistent with reports that CTZ enhances AMPA receptor function by increasing AMPA receptor currents and lengthening single-channel opening, while copper inhibits AMPA receptor function by decreasing these currents [12, 44]. 

An additional aspect regarding the modulation of PPF after LTP was the observation that, in the CA1 region, the PPR was increased by copper, consistent with a mechanism whereby the *P*_*r*_ was decreased. However, after LTP was expressed in the CA3 region, the PPR was decreased, consistent with a mechanism involving an increase in the *P*_*r*_. If PTX, used in the CA3 region, enhanced the *P*_*r*_ by blocking an antagonistic pathway, this could help explain our result. However, presynaptic GABA_A_ receptors facilitate neurotransmitter release, and their inhibition blocks LTP in the CA3 region, so presynaptic GABA_A_ receptors may not have been involved [37]. The *P*_*r*_ is also influenced by the relative sizes of the readily releasable and reserve pools of neurotransmitter, with small releasable pools and large reserve pools supporting paired-pulse facilitation and the converse supporting paired-pulse depression [45]. Thus, the apparent increase in *P*_*r*_ which we observed as a decreased PPR could have resulted from an increase in the readily releasable pool. This would be consistent with a copper-dependent enhancement of vesicular binding to the presynaptic terminal as has been shown in isolated brain synaptic vesicles [34]. Overall, our data clearly shows a copper-dependent modulation of the PPR after LTP in both the CA3 and CA1 hippocampal areas and strongly points to a role for copper in modulating presynaptic plasticity during LTP.

Combined, our work with LTP and short-term pre-synaptic plasticity suggests potential members of a copper interactome that could include both pre- and postsynaptic NMDA receptors, pre-synaptic AMPA, GABA receptors, Rab GTPases, and voltage-gated calcium channels. In addition, the Alzheimer's disease A*β* peptide, the prion protein (PrP^c^), as well as Cu/Zn-superoxide dismutase, are each regulated by binding to copper, have well documented roles in modulating synaptic plasticity, and could be part of a set of copper-interacting proteins that influence the deterioration of learning and memory in neurodegenerative diseases [46–49]. Indeed, copper-based therapies based on an interaction between copper and the Alzheimer's disease A*β* protein are currently in development for the treatment of Alzheimer's disease and show promise for treating prion diseases [49–52]. Thus, the work presented here makes a relevant contribution to our understanding of the mechanism by which copper affects synaptic plasticity and points to its presynaptic involvement in the etiology and treatment of copper-dependent neurodegenerative disorders. 

## 5. Conclusions

We have shown that copper inhibited NMDA receptor-independent LTP in the CA3 region of the mouse hippocampus. Copper had interactions with synaptic plasticity at a presynaptic level, as indicated by our finding that copper significantly enhanced the PPR in the CA1 region and decreased the PPR in the CA3 region in an LTP-dependent manner. In further support of this, copper reduced PTP in the CA1 region and CA3 regions. Thus, LTP caused the appearance of a copper-sensitive factor which modulated the PPR.
